# The prognostic value of systemic vascular resistance in heart failure patients with permanent atrial fibrillation: a retrospective study

**DOI:** 10.1007/s00380-023-02314-0

**Published:** 2023-09-25

**Authors:** Zongpeng Jing, Jingjing Zhang, Jijun Ding, Zongqian Xue

**Affiliations:** Department of Cardiology, Aoyang Hospital Affiliated to Jiangsu University, Zhangjiagang, 215600 China

**Keywords:** Heart failure, Permanent atrial fibrillation, Worsening heart failure, Systemic vascular resistance, Transthoracic impedance cardiography

## Abstract

Heart failure (HF) and permanent atrial fibrillation (AF) interact mutually, exacerbating hemodynamic effects and causing adverse outcomes and increased healthcare costs. Monitoring hemodynamic indicators in patients with these comorbidities is crucial for effective clinical management. Transthoracic impedance cardiography (ICG) has been widely employed in assessing hemodynamic status in clinical settings. Given the limited research on the prognostic significance of ICG parameters in HF with permanent AF, we undertook this study. A total of 66 HF patients with permanent AF were included in this retrospective study, and the primary outcome was rehospitalization due to worsening HF within 180-day post-discharge. Cox regression analysis was performed to explore the connection between ICG-evaluated parameters and the outcome risk. Receiver operating characteristic (ROC) curve analysis determined the optimal cutoff values of risk factors, subsequently applied in plotting Kaplan Meier (KM) survival curves. Multivariate Cox regression analysis revealed that systemic vascular resistance (SVR) both on admission and at discharge independently predicted rehospitalization for worsening HF. ROC analysis established optimal SVR cutoff values: 320.89 (kPa s/L) on admission and 169.94 (kPa s/L) at discharge (sensitivity 70%, specificity 94.4%, area under the curve (AUC) 0.831, respectively, sensitivity 90%, specificity 55.6%, AUC 0.742). KM survival curves analysis showed that patients with SVR > 320.89 (kPa s/L) on admission had an 8.14-fold (*P* < 0.001) increased risk of the end-point event compared with those with SVR ≤ 320.89 (kPa s/L). Similarly, patients with SVR > 169.94 (kPa s/L) at discharge faced a risk elevated by 6.57 times (*P* = 0.002) relative to those with SVR ≤ 169.94 (kPa s/L). In HF patients with permanent AF, SVR measured by ICG emerges as an independent risk factor and clinical predictor for HF deterioration-related readmission within 180 days after discharge. Higher SVR levels, both upon admission and at discharge, correlate with an incremental rehospitalization risk.

## Introduction

Heart failure (HF) has been a growing public health issue. Despite marked reductions in HF-related mortality rates, rehospitalization owing to recurrent HF deterioration remains prevalent worldwide. Some studies [1, 2] have indicated that approximately 50% of HF patients experienced readmission within 6 months after discharge, with 70% of these cases linked to known HF exacerbation [3]. Atrial fibrillation (AF) has been the most common persistent arrhythmia in HF, impacting around 25% of patients on average, with its incidence rising [4]. HF and AF mutually worsen each other, leading to higher risks of death and readmission after discharge [5, 6], imposing a substantial burden on healthcare systems.

Inflammation, oxidative stress, and neuroendocrine abnormalities related to HF contribute to AF while ongoing AF worsens left ventricular function and HF progression. HF triggers increased left atrial pressure, affecting atrial function and causing hemodynamic imbalance [7, 8], raising hospitalization and all-cause mortality [9–11]. Considering that disrupted hemodynamics by HF and AF result in unfavorable results, understanding the hemodynamic state of patients with these comorbidities is essential and holds clinical value.

The Swan–Ganz floating catheter [12] is globally acknowledged as the "Gold Standard" for determining hemodynamic status. However, it involves invasive procedures with demanding technical prerequisites, complication rates ranging between 3 and 5% [13, 14], and high costs. It is clinically used in critically ill patients. As a non-invasive hemodynamic monitoring method, transthoracic impedance cardiography (ICG) can conveniently and comprehensively detect hemodynamic data, understand the immediate hemodynamic changes, and provide objective and quantitative indicators. Relevant studies have verified the accuracy of ICG [15–20].

In recent years, ICG has played an essential role in guiding medical therapy for acute and chronic HF [21–23], in clinical drug trials [24], in the evaluation of the effectiveness of other treatments for HF [25], in aiding medical care [26], and in monitoring during AF ablation and examining postoperative effects [27, 28]. However, there is a lack of studies on using ICG variables to judge the prognosis of patients with HF and AF, which prompted us to undertake this study.

## Methods

### Study population

**Fig. 1:**
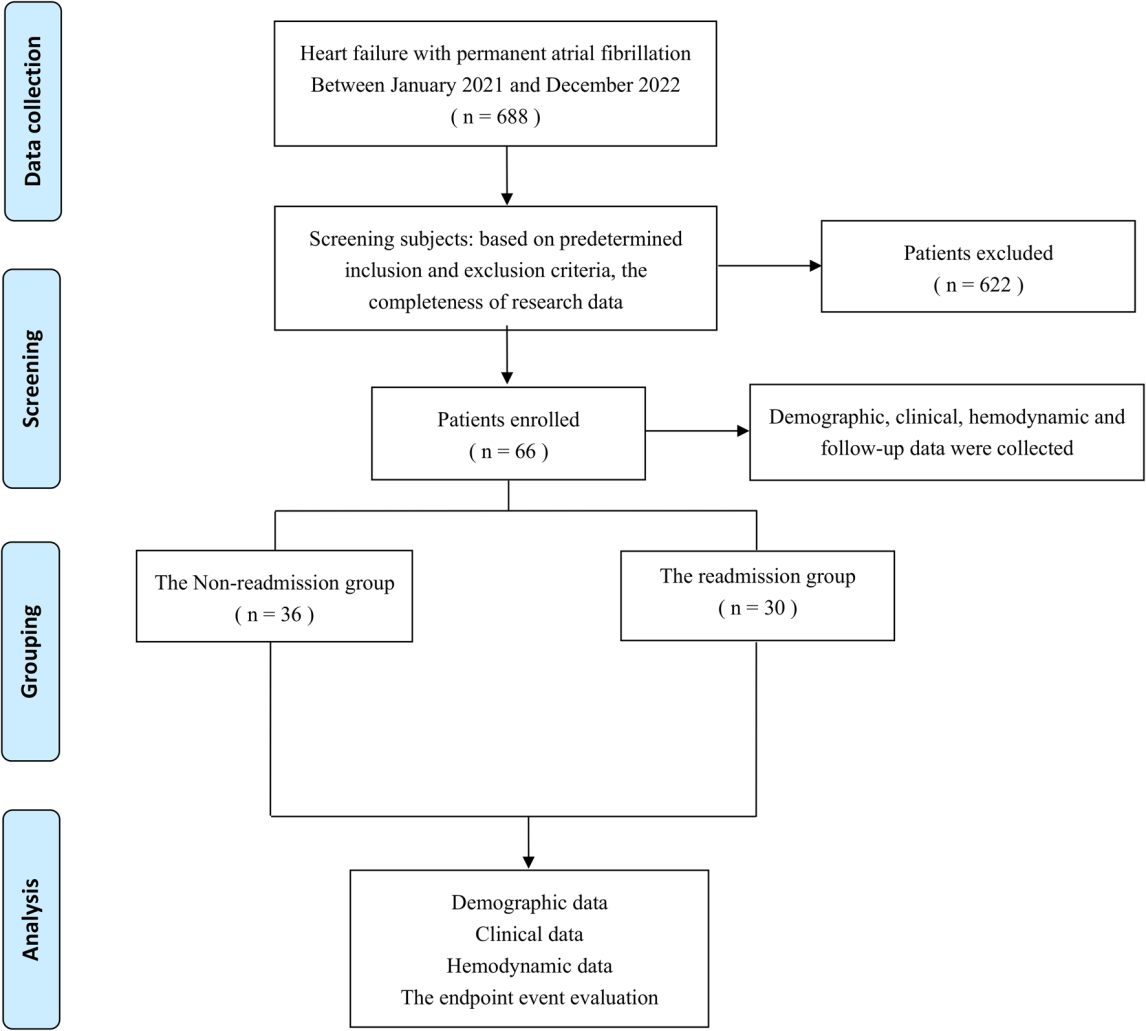
Study design and procedures

We reviewed the patients with HF and permanent AF admitted to the Cardiology Department of Aoyang Hospital Affiliated to Jiangsu University from January 2021 to December 2022 and ultimately included 66 subjects (Fig. 1). HF and permanent AF criteria meet the European Society of Cardiology guideline definitions [29, 30].

Exclusion criteria: body weight below 40 kg or above 100 kg, inability to cooperate as a result of mental and psychological abnormalities, pacemaker implantation, skin ulceration of chest wall, Second-Degree Type II or Third-Degree atrioventricular block, acute infectious or autoimmune diseases in the acute stage, hyperthyroidism, acute coronary syndrome, hypertrophic obstructive cardiomyopathy, large arteritis, aortic aneurysm, severe peripheral vascular disease, dialysis status, severe valve stenosis or regurgitation, congenital heart disease, severe pulmonary hypertension, acute pulmonary embolism, constrictive pericarditis, massive pleural or pericardial effusion, pneumothorax, malignancy, shock status, severe anemia, cachexia.

Subjects were categorized into the readmission and non-admission groups based on rehospitalization due to worsening HF within a 180-day follow-up period after discharge. Readmission for HF deterioration was the end-point event. All participants included were discharged with clinical improvement, defined as stable vital signs, alleviation of symptoms and signs of circulatory congestion, and no need for intravenous drug management. The criteria for worsening HF: symptoms and signs accompanied by circulatory congestion, New York Heart Association (NYHA) classification of cardiac function ≥ 3, and no improvement in symptoms with oral pharmacotherapy. This study was approved by the Medical Ethics Committee of our institution ((2021) Ethics Approval No. 010), and written informed consent was waived because of the retrospective nature.

### Data collection and follow-up

The following data were collected through the hospital database: demographic information, medical history, physical examination, blood tests, including N-terminal pro-B-type natriuretic peptide (NT-pro BNP), high-sensitivity cardiac troponin (HS-cTn), creatinine clearance evaluated by Cockcroft–Gault formula, total bilirubin, serum albumin, hemoglobin, D-Dimer, thyroid-stimulating hormone (TSH), blood sodium, serum total cholesterol, serum triglyceride, low-density lipoprotein. Arterial pressure, rhythm type, mean ventricular rate determined by 24-h ambulatory monitoring, left atrial transverse diameter, left atrial volume index, left ventricular end-diastolic diameter, left ventricular ejection fraction (LVEF) measured by transthoracic echocardiography, and medication prescriptions at discharge were gathered. The post-discharge end-point event was collected through retrospective medical records until December 2022. Data were checked and entered collaboratively by two investigators.

### Transthoracic impedance cardiography (ICG)

**Fig. 2:**
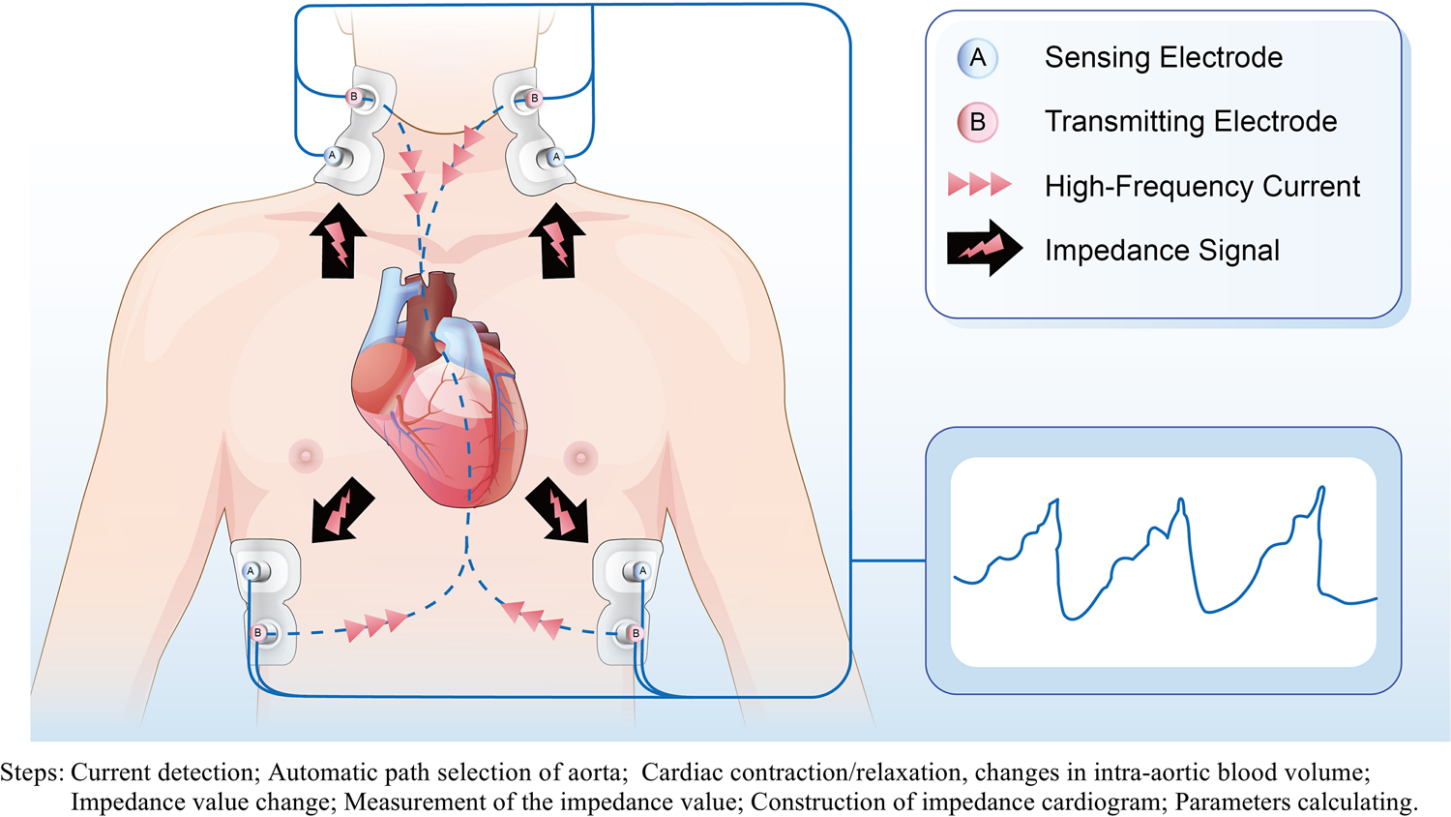
Technical principle of ICG: Ohm’s law

ICG is non-invasive, relying on varying electrical impedance in various tissues and water contents of the human body. In one cardiac cycle, impedance value changes with the blood volume and flow rate in thoracic vessels. Analyzing thoracic impedance shifts help determine hemodynamic parameters of blood movement [31]. Notably, the procedure is simple, requiring four pairs of electrodes on the neck and chest. Two pairs generate signals, while the other two detect them. After thoracic tissue rectification, instant signal changes can be observed (Fig. 2). In this study, nine clinically significant hemodynamic variables obtained by the non-invasive Hemodynamic Monitoring System (CSM3000, Qianfan Medical Co., Ltd, China) were defined as follows (Table 1).

**Table 1:** Definition of hemodynamic parameters obtained by transthoracic impedance cardiography (ICG)

ICG variable	Definition
Cardiac index (CI)	Cardiac output per unit of body surface area to evaluate cardiac pumping function
Chronotropy	Representing the regulatory ability of the autonomic nerve to adjust the heart rate in compliance with the changes in the cardiac index
Thoracic fluid conductivity (TFC)	The indicator to reflect the increase of pleural fluid
Stroke volume variation (SVV)	A parameter to evaluate the distribution of pleural fluid together with TFC and Volemia
Volemia	The intravascular circulation blood volume to estimate the distribution of pleural fluid together with SVV and TFC
Systemic vascular resistance (SVR)	The average total resistance per minute of peripheral blood vessels
Left ventricular stroke work (LSW)	The work done by one left ventricular contraction
Preejection phase (PEP)	The time from the mitral valve's closure to the aortic valve's opening and the process of increasing the ventricular pressure for myocardial contraction
Left ventricular ejection time (LVET)	The time from the opening of the aortic valve and the injection of left ventricular blood into the aorta to the closing of the aortic valve

### Statistical analysis

Continuous variables with normal distribution were expressed as means ± standard deviation, while non-normally distributed variables as medians and interquartile ranges (IQRs); categorical variables were presented as frequency and percentage (%). Statistical tests included the *t* test for continuous variables, the Chi-square or Fisher’s exact test for categorical variables, and the Mann–Whitney U test for non-normally distributed variables. Cox regression analysis examined the relationship between the end-point event and parameters. Variables (*P* < 0.05) were included in multivariate analysis. Receiver-operating characteristic (ROC) curves established cutoff values for rehospitalization prediction. Kaplan–Meier (KM) analysis assessed the prognostic value, and significance was set at *P* < 0.05. Data were analyzed using SPSS version 25 and plotted with GraphPad Prism version 9.3 and R version 4.2.2.

## Results

### Baseline characteristics

The study included 66 HF patients with permanent AF. Of these, 30 with worsening HF were readmitted within 180 days (readmission group), while 36 were not (non-admission group, Table 2). No significant differences were found in age, gender, and BMI between the two groups. Mean ventricular rate, systolic, and diastolic blood pressure showed no group variations. Clinical conditions (smoking, alcohol, hypertension, diabetes, stroke history, CAD/MI history, PCI/CABG history, left bundle branch block, and right bundle branch block) were similar. Blood test parameters (NT-pro BNP, HS-cTn, etc.) did not differ significantly. Echocardiography measurements remained without significant differentiation.

**Table 2:** Characteristics of the study subjects

	All subjects	Readmission	Non-readmission	*P* value
	*N* = 66	*N* = 30	*N* = 36	
Age, years	71.97 ± 9.27	70.77 ± 8.87	72.97 ± 9.61	0.340
Male/female, *n*	34/32	15/15	19/17	0.822
BMI, kg/m^2^	24.44 (3.85)	24.02 (5.22)	24.73 (3.11)	0.704
Heart rate, beat/min	78.50 (27)	73 (29)	81.50 (25)	0.070
Systolic blood pressure, mmHg	120.58 ± 15.35	124.37 ± 17.53	117.42 ± 12.67	0.067
Diastolic blood pressure, mmHg	72.08 ± 11.86	74.50 ± 11.58	70.06 ± 11.87	0.131
Preexisting clinical conditions				
Smoking, *n* (%)	15 (23)	7 (23)	8 (22)	0.915
Alcohol, *n* (%)	11 (17)	5 (17)	6 (17)	> 0.999
Hypertension, *n* (%)	44 (67)	21 (70)	23 (64)	0.600
Diabetes, *n* (%)	13 (20)	7 (23)	6 (17)	0.498
Stroke history, *n* (%)	4 (6)	2 (7)	2 (6)	> 0.999
CAD/MI history, *n* (%)	19 (29)	9 (30)	10 (28)	0.843
PCI/CABG history, *n* (%)	5 (8)	1 (3)	4 (11)	0.470
Left bundle branch block, *n* (%)	3 (5)	2 (7)	1 (3)	0.871
Right bundle branch block, *n* (%)	4 (6)	1 (3)	3 (8)	0.742
Blood test results				
NT-pro BNP, pg/mL	2137.25 (3080.50)	2181.70 (2394)	2128.05 (3278.30)	0.787
HS-cTn, ng/L	12 (25)	12.50 (28.30)	11.50 (23)	0.827
Creatinine clearance, mL/min	70.02 ± 27.33	70.63 ± 24.52	69.51 ± 29.81	0.870
Total bilirubin, umol/L	22.45 (14)	25.45 (16.50)	20.25 (10.90)	0.254
Serum albumin, g/L	40.10 (3.20)	40.25 (4.30)	40.05 (3.20)	0.892
Hemoglobin, g/L	134.98 ± 17.24	134.57 ± 13.98	135.33 ± 19.74	0.859
D-dimer, mg/L	0.47 (0.80)	0.43 (0.69)	0.49 (0.87)	0.842
TSH, mIU/L	2.14 (1.86)	2.24 (2.25)	2.03 (1.81)	0.995
Blood sodium, mmol/L	139.80 (3.70)	139.55 (3)	140 (4.20)	0.772
Serum total cholesterol, mmol/L	3.83 (1.22)	3.65 (1.43)	3.93 (1.25)	0.185
Serum triglyceride, mmol/L	1.04 (0.73)	0.95 (0.72)	1.08 (0.62)	0.172
Low-density lipoprotein, mmol/L	2.22 (1.05)	2.18 (1.02)	2.36 (1.03)	0.309
Echocardiographic findings				
Left atrial transverse diameter, mm	44 (6)	45 (8)	46 (6)	0.131
Left atrial volume index, mL/m^2^	50.30 (18.40)	50.60 (19.90)	48.30 (14.40)	0.205
Left ventricular end-diastolic diameter, mm	51.36 ± 6.85	51.87 ± 6.88	50.94 ± 6.90	0.590
Left ventricular ejection fraction, %	51.80 (18.80)	48.50 (19.50)	54 (18.30)	0.420

Data are expressed as mean ± SD, or median (interquartile range), or number of patients (%)

*BMI* body mass index, *CAD* coronary artery disease, *MI* myocardial infarction, *NT-proBNP* N-terminal pro-B-type natriuretic peptide, *HS-cTn* high-sensitivity cardiac troponin, *TSH* thyroid-stimulating hormone, *PCI* percutaneous coronary intervention, *CABG* coronary artery bypass grafting

### Medication administration at discharge

At discharge, the drug prescriptions of β-blocks, loop diuretics, Valsartan/Sacubitril, SGLT2i, ACEI/Sartans, aldosterone antagonists, Nitrates, Digoxin, Statins, antiplatelet drugs, oral anticoagulants, Diltiazem or Verapamil, Propafenone, Amiodarone, between the two groups were no statistical differences (Table 3).

**Table 3:** Differences in prescriptions at discharge

Medications at discharge	All subjects	Readmission	Non-readmission	*P* value
	*N* = 66	*N* = 30	*N* = 36	
β-Blocks, *n* (%)	51 (77)	22 (73)	29 (81)	0.486
Loop diuretics, *n* (%)	46 (70)	23 (77)	23 (64)	0.261
Valsartan/sacubitril, *n* (%)	23 (35)	14 (47)	9 (25)	0.066
SGLT2i, *n* (%)	8 (12)	2 (7)	6 (17)	0.389
ACEi/sartans, *n* (%)	20 (30)	6 (20)	14 (39)	0.096
Aldosterone antagonists, *n* (%)	46 (70)	23 (77)	23 (64)	0.261
Nitrates, *n* (%)	6 (9)	2 (7)	4 (11)	0.845
Digoxin, *n* (%)	24 (36)	9 (30)	15 (42)	0.327
Statins, *n* (%)	35 (53)	15 (50)	20 (56)	0.652
Antiplatelet drugs, *n* (%)	10 (15)	4 (13)	6 (17)	0.975
Oral anticoagulants, *n* (%)	49 (74)	22 (73)	27 (75)	0.877
Diltiazem or verapamil, *n* (%)	1 (2)	0 (0)	1 (3)	> 0.999
Propafenone, *n* (%)	0 (0)	0 (0)	0 (0)	
Amiodarone, *n* (%)	0 (0)	0 (0)	0 (0)	
D-CCB, *n* (%)	7 (11)	3 (10)	4 (11)	> 0.999

Data are expressed as number of patients (%)

*D-CCB* dihydropyridine calcium channel blockers, *SGLT2i* sodium–glucose cotransporter-2 inhibitors, *ACEI* angiotension converting enzyme inhibitors

### Relationship between ICG parameters and the rehospitalization event

As shown in Table 4, univariate Cox regression analysis showed that the occurrence of rehospitalization due to worsening HF was significantly correlated with SVR, Chronotropy, and CI on admission: SVR (HR: 1.007, 95% CI 1.005–1.010, *P* < 0.001), Chronotropy (HR: 0.995, 95% CI 0.994–0.997, *P* < 0.001), CI (HR: 0.180, 95% CI 0.064–0.512, *P* = 0.001), and multivariate analysis confirmed the unique efficacy of SVR (HR: 1.012, 95% CI 1.006–1.018, *P* < 0.001). In addition, SVR at discharge was significantly associated with the end-point event (HR: 1.004, 95% CI 1.002–1.006, *P* < 0.001 and HR: 1.004, 95% CI 1.000–1.008, *P* = 0.041, respectively), even after adjusting for the significant variable CI in univariate analysis (HR: 0.452, 95% CI 0.251–0.814, *P* = 0.008).

**Table 4:** Relationship between readmission risk and hemodynamic data by COX regression analysis

Variables	Univariate analysis		Multivariate analysis	
	HR (95% CI)	*P* value	HR (95% CI)	*P* value
On admission				
Cardiac index, L/min m^2^	0.180 (0.064–0.512)	**0.001**	1.314 (0.401–4.306)	0.652
Chronotropy, %	0.995 (0.994–0.997)	**< 0.001**	1.004 (0.999–1.008)	0.114
Thoracic fluid conductivity, 1/kΩ	1.038 (0.998–1.080)	0.062		
Stroke volume variation, %	1.005 (0.984–1.025)	0.648		
Volemia, %	1.001 (0.998–1.004)	0.608		
Systemic vascular resistance, kPa s/L	1.007 (1.005–1.010)	**< 0.001**	1.012 (1.006–1.018)	**< 0.001**
Left ventricular stroke work, g.m/beat	0.978 (0.952–1.004)	0.094		
Preejection phase, ms	1.003 (0.980–1.027)	0.790		
Left ventricular ejection time, ms	1.008 (0.996–1.021)	0.971		
At discharge				
Cardiac index, L/min m^2^	0.452 (0.251–0.814)	**0.008**	0.978 (0.399–2.399)	0.961
Chronotropy, %	0.998 (0.996–1.000)	0.062		
Thoracic fluid conductivity, 1/kΩ	1.020 (0.975–1.066)	0.395		
Stroke volume variation, %	1.004 (0.977–1.031)	0.789		
Volemia, %	1.001 (0.998–1.003)	0.581		
Systemic vascular resistance, kPa s/L	1.004 (1.002–1.006)	**< 0.001**	1.004 (1.000–1.008)	**0.041**
Left ventricular stroke work, g m/beat	0.994 (0.975–1.013)	0.531		
Preejection phase, ms	1.019 (0.993–1.046)	0.147		
Left ventricular ejection time, ms	1.003 (0.991–1.017)	0.601		

Variables with *P* < 0.05 are considered significant and are indicated in bold

*HR* hazard ratio, *CI* confidence interval

### The value of SVR for predicting the end-point event

**Fig. 3:**
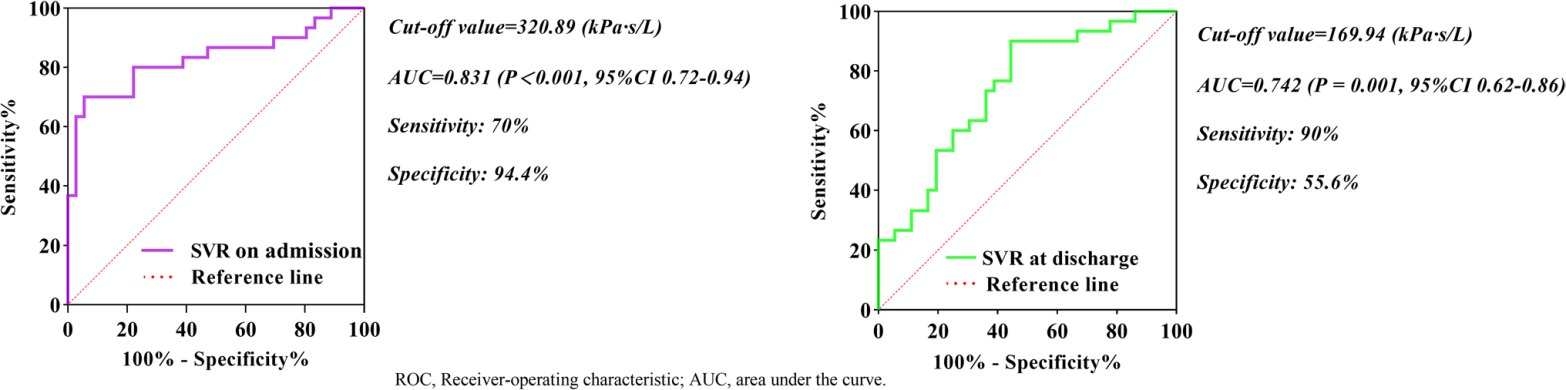
ROC analysis of the SVR as a predictor of readmission risk

**Fig. 4:**
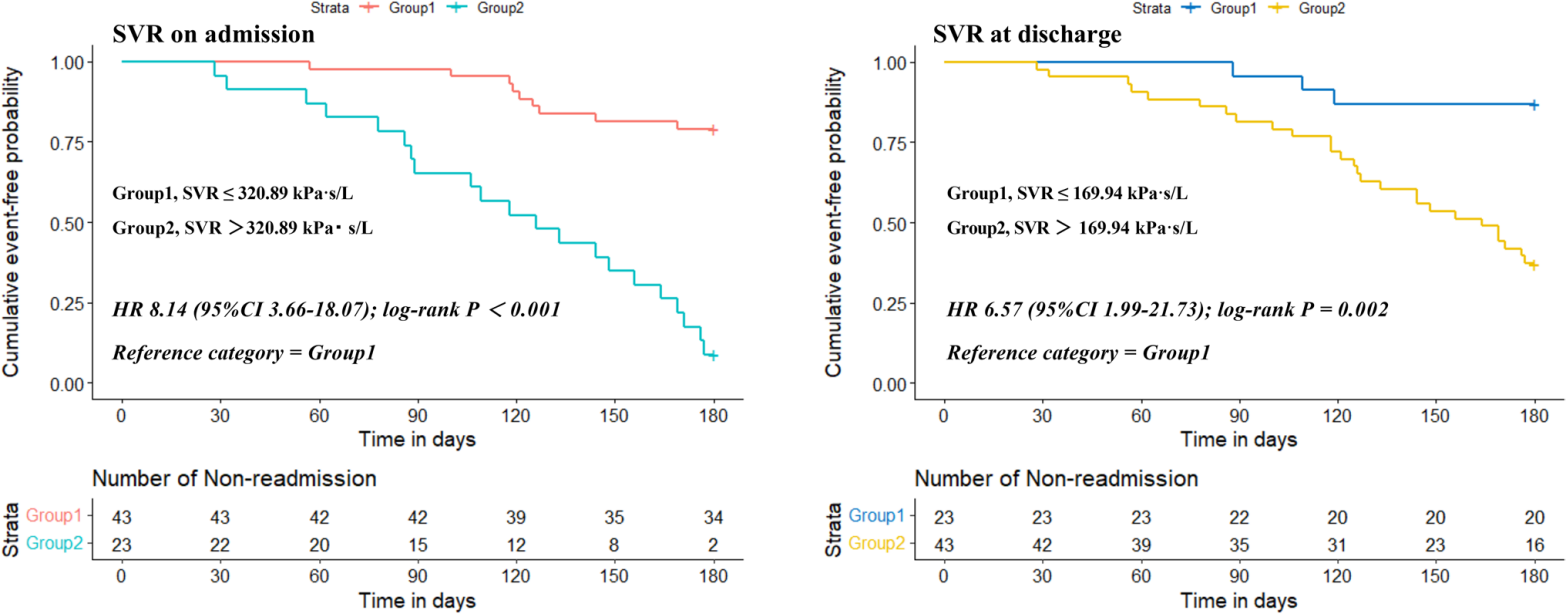
Kaplan–Meier curve of the endpoint event

ROC analysis of the SVR to predict readmission for HF exacerbation revealed (Fig. 3) that the SVR value 320.89 (kPa s/L) on admission was the best cutoff level for predicting rehospitalization, which gave 70% sensitivity and 94.4% specificity with an area under the curve (AUC) of 0.831 (95% CI 0.72–0.94, *P* < 0.001). Meanwhile, employing the designated cutoff value to divide subjects into distinct groups, the KM survival analysis was performed using the log-rank test (Fig. 4). Patients with SVR > 320.89 (kPa s/L) on admission had an 8.14-fold increased risk of the end-point event (95% CI 3.66–18.07, *P* < 0.001) compared with those with SVR ≤ 320.89 (kPa s/L). Interestingly, an SVR value of 169.94 (kPa s/L) at discharge was the optimal cutoff level to predict rehospitalization for worsening HF, presenting 90% sensitivity and 55.6% specificity with an AUC of 0.742 (95% CI 0.62–0.86, *P* = 0.001). Survival analysis suggested that patients with SVR > 169.94 (kPa s/L) at discharge faced a 6.57-fold increased risk of the end-point event (95% CI 1.99–21.73, *P* = 0.002) in contrast to those with SVR ≤ 169.94 (kPa s/L).

## Discussion

This study found that SVR, both on admission and at discharge, emerged as an independent risk factor and predictor of rehospitalization for worsening HF within 180-day post-discharge in HF patients with permanent AF. Moreover, patients with elevated SVR faced an increased risk of readmission stemming from HF aggravation within this period. To the best of our knowledge, this is the first study demonstrating the utility of ICG parameter SVR as a straightforward marker and predictive risk factor for 180-day rehospitalization in patients with HF and permanent AF.

### Clinical predictors of rehospitalization in HF and AF

Reducing rehospitalization for HF and AF patients is vital for better outcomes and cost control. For this purpose, it is essential to identify high-risk groups and implement interventions. Currently, there is an ongoing inquiry into the rehospitalization risk for HF and AF patients. However, compared to the previous studies [32–35], new findings still emphasize sociodemographic [36, 37], clinical tests [38, 39], comorbidities [40, 41], medical regimens and quality of care [42], and risk models [43–46]. These outcomes identified risk factors at diffident time intervals (30, 90, 180, or ≥ 365 days). In addition to differences in study durations, distinctions in participant demographics, health status, and research methods result in incomparable data. Moreover, due to an uncertain balance between medical and non-medical factors, the multi-marker prediction models for HF and AF readmission may not be optimally accurate. In addition, the complexity and limited availability of these factors impact their objective evaluation and clinical applicability. Unlike prior studies, our research centers on a crucial pathophysiological mechanism of HF deterioration, hemodynamic imbalance unexplored in other investigations. Of importance, this methodology holds promise because of its convenience, scientific rigor, objectivity, and reproducibility.

### Prognostic value of ICG parameters in HF and AF

Limited research has explored the relationship between non-invasive hemodynamic parameters and the prognosis of HF and AF. Some studies have focused on cardiac death as the end-point event. For instance, Andrius et al. reported [47] that chronic HF patients with TFC ≥ 36.91/kΩ had a 4.6-fold higher risk of cardiac death within 36 months of follow-up. Similarly, ICU-admitted acute HF patients with TFC ≥ 34.1/kΩ faced increased 6-month mortality [48]. However, these studies did not account for SVR and specify AF presence. Another investigation [49] noted higher mortality at 1 and 4 years in systolic HF patients with BN* P* ≥ 450 pg/ml and TFC ≥ 40.1/kΩ, excluding AF patients. Hao‐Chih et al. [50] linked exponential TFC ≥ 0.5/kΩ/m^2^ to increased HF readmission and all-cause death based on nocturnal impedance measurements. The observation did not include SVR, affecting comparability. A cohort study [51] tied exponential TFC and LVET measured by ICG to HF events within 14 days. Yet, it lacked data beyond this period and was underpowered to assess the predictive value of ICG over 14 days. Notably, participants with no improvements in HF symptoms within 7 days of treatment and those planned for intravenous medications (diuretics, vasodilators, or inotropic agents) were excluded, making comparisons with our study inconclusive. Hence, differences in study participants, parameters, and outcomes prevent direct comparison of prognostic implications of ICG parameters in HF and AF.

### Prognosis value of SVR in HF and AF

Dynamic changes in vascular tone are another critical component in worsening HF pathogenesis. Heightened sympathetic activation and vasoconstrictor substances release, common in deteriorating HF, intensify arterial constriction, then increase SVR. Increased afterload triggers a rise in left ventricular pressure, enhancing ventricular wall stress, worsening myocardial ischemia, causing myocardial injury, deteriorating left ventricular pump performance, and elevating the likelihood of severe cardiac events [52–54]. Teerlink et al. [55] found novel vasodilator agents stabilize HF patients’ hemodynamic balance by improving SVR, reducing HF deterioration and mortality risk. It is speculated that vasodilators targeting vascular resistance pathways hold promise for treating HF deterioration [56]. In an investigation [57] of HF patients during 1-month outpatient follow-up after improvement and discharge, hemodynamic indices by whole-body impedance measurement examined the rehospitalization risk for HF aggravation. Univariate analysis indicated that higher SVR predicted HF rehospitalization (100% sensitivity, 68.6% specificity, and 0.89 AUC), but the multivariate analysis found no interplay. Conversely, our findings suggested that SVR admission value had better specificity, while discharge value had good sensitivity. This variation comes from research design: their patients were younger, with better cardiac status, lower proportion of AF and baseline SVR than ours, not technology [58]. In addition, a multi-center prospective cohort study [59] analyzed SVR via ultrasound electrocardiogram. It focused on coronary heart disease patients, dividing them into SVR tertiles: < 5.6, 5.6–6.9, and ≥ 6.9. Over a 5-year follow-up, the ≥ 6.9 group had higher cardiovascular risks. This study differs from ours in design, included population, SVR measurement (direct correlation between this approach and invasive one has never been verified), and end-points. Nevertheless, its conclusions and ours endorse the significance of high SVR levels as a crucial risk factor in worsening HF and predicting adverse cardiovascular outcomes.

### SVR and underlying cardiac diseases

SVR pertains to the level of hindrance encountered by blood flow within the vessels of the circulatory system. The elevation of SVR stems from a multifaceted interplay of factors, including vasoconstriction, heightened vascular wall thickness, augmented blood viscosity, reduced vascular elasticity, vascular endothelial dysfunction, disturbances in the neuroendocrine system, and inflammatory responses within the vascular wall, among others. These underlying pathophysiological mechanisms may interact to contribute to the escalation of SVR.

Cardiovascular diseases directly or indirectly raise SVR by affecting these mechanisms. For instance, the diminished cardiac function activates the sympathetic nervous and renin–angiotensin–aldosterone systems, causing vasoconstriction, water retention, and increased blood volume, elevating resistance [60]. Hypertension leads to peripheral vasoconstriction and vascular remodeling, narrowing arterial diameter and obstructing blood flow [61].

In our study, the exclusion of various hemodynamically impactful diseases and the presence of comparable baseline data, including blood pressure and relevant resistance-affecting medications, minimized external influences on SVR, enabling a direct evaluation of the link between HF with AF and SVR. Since we did not specify the cardiac etiology of the enrolled population, we cannot ascertain whether different cardiac causes might affect SVR measurement in our study. Further research on this aspect would also hold significant value.

Our conclusions quantified the linkage between SVR and rehospitalization within 180 days for HF patients with permanent AF. This insight may aid clinicians in identifying high-risk readmissions, optimizing treatment plans, strengthening outpatient follow-up, and even ultimately reducing adverse events—an essential contribution of this study.

### Limitations and future research directions

This study has limitations: small sample size, all Chinese participants, HF types, some comorbidities excluded, and retrospective design impact generalization. Prospective multi-center research with a large sample is needed to further validate the prognostic value of SVR in different HF and permanent AF cases.

## Conclusions

In conclusion, our findings show a strong link between elevated SVR measured by ICG and a 180-day readmission risk for worsening HF in cardiac insufficiency patients with permanent AF.

## Data Availability

The datasets generated and analyzed during the current study are available from the corresponding author on reasonable request.
